# Photochemistry of Aromatic N‐Oxides in Water Probed by Time‐Resolved X‐ray Absorption Spectroscopy

**DOI:** 10.1002/chem.202502775

**Published:** 2025-11-29

**Authors:** Maximilian Paradiz Domínguez, Robby Büchner, Mattis Fondell, Albert M. Brouwer

**Affiliations:** ^1^ Van ’t Hoff Institute for Molecular Sciences University of Amsterdam Amsterdam Netherlands; ^2^ Institute for Methods and Instrumentation for Synchrotron Radiation Research Helmholtz‐Zentrum Berlin für Materialien Und Energie Berlin Germany

**Keywords:** density functional calculations, nitrogen heterocycles, photochemistry, time‐resolved spectroscopy, X‐ray absorption spectroscopy

## Abstract

Aromatic N‐oxides have a rich photochemistry, but the primary steps have not been investigated in much detail. In this work pyridine N‐oxide and pyridazine N‐oxide are studied using steady‐state and time‐resolved X‐ray absorption spectroscopy (XAS). Absorption changes at the nitrogen and oxygen K‐edges following UV photoexcitation are recorded on a timescale of picoseconds to hundreds of nanoseconds. The spectral features are assigned to the presence of specific transient intermediates. Quantum chemical calculations indicate that the excited state dynamics in the S_1_ state are characterized by a fast deplanarization. After reaching a minimum energy crossing point (MECP), evolution on the ground state surface leads to the starting materials and metastable products. The primary photoproduct of pyridine N‐oxide, oxaziridine **3**, is stable on the sub‐microsecond timescale. In the photochemistry of pyridazine N‐oxide, oxaziridine and oxadiazepine intermediates are not observed, and (*Z*)‐4‐diazobut‐2‐enal is formed within 100 ps. The mechanistic details of the two different N‐oxides uncovered through characteristic features in the near‐edge X‐ray absorption fine structure (NEXAFS) region serve as an example of how time‐resolved XAS enables the characterization of photochemical dynamics beyond those accessible to more traditional spectroscopies.

## Graphical abstract

Time‐resolved XAS at the nitrogen and oxygen K‐edges is applied to the photorearrangement of two N‐oxides in water. Pyridine N‐oxide rapidly forms the oxaziridine isomer. Pyridazine N‐oxide undergoes ring opening to the diazo compound, without any evidence of intermediates.

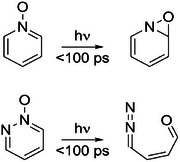

## 1 Introduction

**FIGURE 1:**
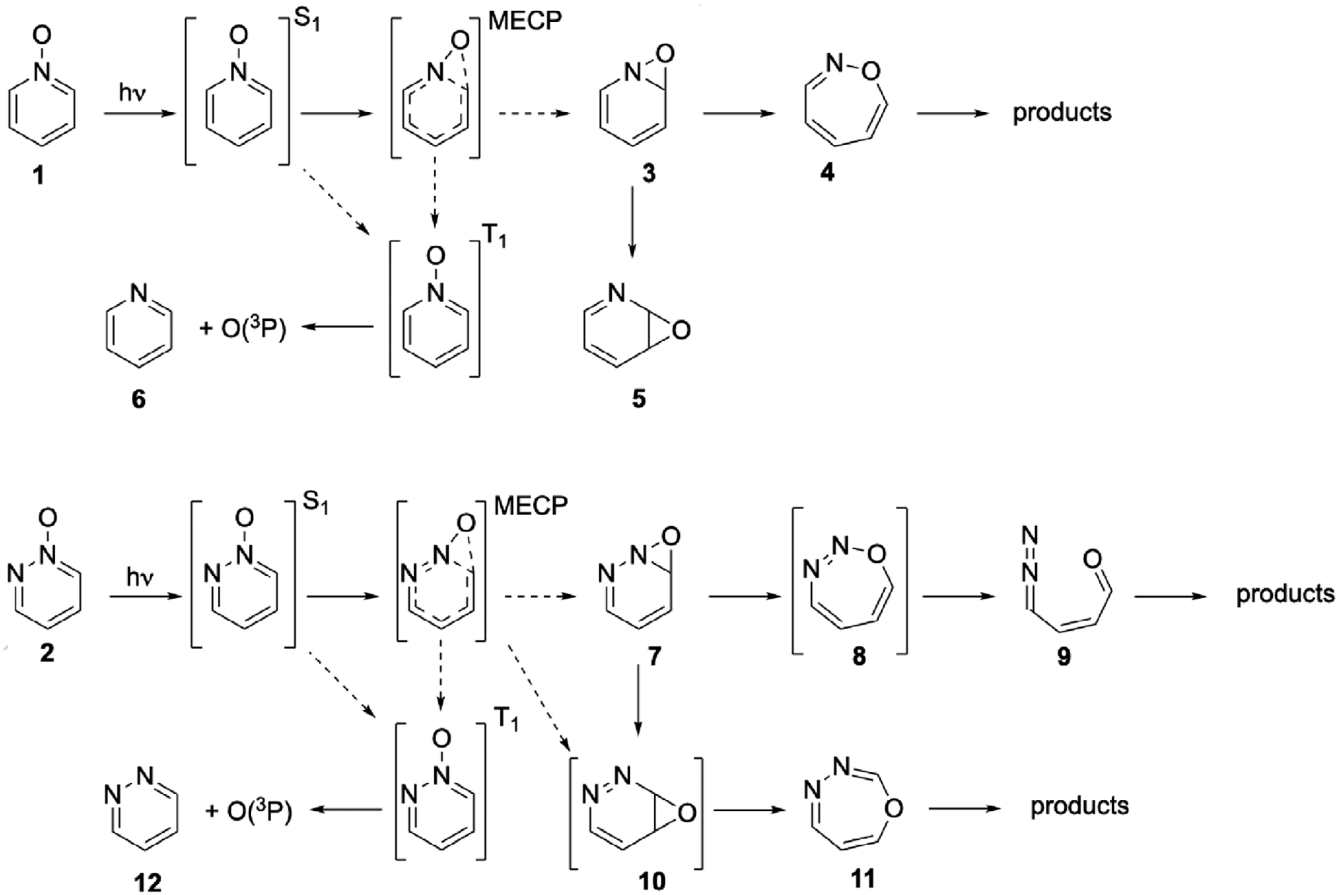
Chemical structures of pyridine N‐oxide **1** and pyridazine N‐oxide **2**, and schemes of the photochemistry probed in the present study. The dotted arrows indicate nonradiative transitions.

The photochemistry of aromatic N‐oxides, of which pyridine N‐oxide (compound **1** in Figure 1) is one of the simplest representatives, has been investigated over many years and has been the subject of multiple reviews [1‑4]. The formation of an oxaziridine (**3** in Figure 1) in the first photochemical reaction step was suggested by several authors during the second half of the 1960s [1, 5, 6, 7, 8, 9, 10]. This hypothesis was motivated by the isolation of oxaziridines from the irradiation of nitrones [11] and further supported by the photochemical synthesis of the 1a*H*‐Oxazirino[2,3‐*a*]quinoxalines from quinoxaline 1‐oxides [6]. More recently, it was shown that a photoinduced ring walk of the O atom along the pyridine ring can lead to selective 3‐hydroxylation [12, 13]. Furthermore, a facile 6π electrocyclic ring opening of the oxaziridine leads to oxazepine **4**.

While the oxazepine intermediate from pyridine N‐oxide has not been observed, there are examples in which substituted pyridine N‐oxides produce oxazepines that can be isolated [14, 15]. An example can be seen in the photorearrangement of [8]‐2,6‐pyridinophane N‐oxide [16]. In most cases, the oxazepine is not stable, and breaking of the N‐O bond leads to further products, among which are pyrrole aldehyde derivatives [1, 5, 17]. The identity of the open‐chain intermediates is still under debate, with some articles suggesting that they are singlet vinyl nitrene biradicals [4].

In the gas phase, the main photoproduct of pyridine N‐oxide **1** has been reported to be pyridine. Under these conditions, the deoxygenation was proposed to occur from the triplet state and to require thermal activation [18]. Photoinduced deoxygenation of N‐oxides also occurs in solution. Irradiation of a solution of **1** in benzene with a high‐pressure mercury lamp results in the formation of phenol and pyridine in a yield of 15% [19]. In acetonitrile, the results of flash photolysis experiments were used to argue that O(^3^P) is produced from the triplet state of pyridine N‐oxide in a “modest” quantum yield [20]. On the other hand, 4‐benzoylpyridine N‐oxide undergoes efficient intersystem crossing and forms O(^3^P), but not from the triplet state [21]. Pyridazine N‐oxide **2** has been reported to give rise to photooxygenation of hydrocarbon substrates [22], and also other N‐oxides generate reactive oxygen species upon photoexcitation [23, 24]. The efficiency of oxygen generation is usually low, but aromatic N‐oxides have been developed as a chemically useful source of atomic oxygen in organic solvents [25, 26]. To the best of our knowledge, in these studies oxygen atoms were not directly observed, but only indirectly, through reaction products.

The reactive and photoactive nature of the photoproducts of aromatic N‐oxides can lead to a complex mixture of products upon illumination with UV light. Despite this complexity, useful synthetic applications of N‐oxide photochemistry continue to emerge [16, 26‑30]. This makes an understanding of the primary photoreaction steps an important goal, even 7 decades after the first reports on the topic.

The first steps in the photochemistry of some aromatic N‐oxides have been investigated by means of time‐resolved spectroscopy [31, 32], but the parent compound **1** was not addressed. A likely reason is the requirement of short‐wavelength UV excitation, which is not commonly available, and the lack of strong visible absorption by the photochemical intermediates. We therefore chose to apply a relatively novel technique in photochemistry, namely time‐resolved soft X‐ray absorption spectroscopy (XAS) [33]. For pyridazine N‐oxide **2** time‐resolved visible and infrared spectroscopy and computational quantum chemistry have been reported by Ma et al. [32]. We included **2** in our study because having these data available helps to interpret the time‐resolved XAS results. Ma et al. concluded that in acetonitrile oxaziridine **7** is the primary photoproduct, and it undergoes ring opening to the diazo compound **9**, presumably via **8**, which was not detected. In addition, evidence was presented for the intermediate **11**, although it is unclear how this is formed. The involvement of the triplet state and oxygen atom formation were ruled out in the study of Ma et al., but we added them to Figure 1 because intersystem crossing and oxygen atom formation have been reported for pyridine N‐oxide **1** [19, 20], and pyridazine N‐oxide **2** is known to act as a photooxygenation agent [22, 26].

XAS probes transitions in which core electrons are promoted to vacant molecular orbitals (in the “near‐edge” energy region) and to the ionization continuum (above the edge) [33‑38]. Using tunable sources of X‐rays, absorption spectra can be measured (often detected indirectly, via the emitted photoelectrons) for different core levels of different elements across the periodic table. The ability to probe electronic states in an atom‐specific manner makes XAS a versatile tool in inorganic chemistry [39‑43], but applications to organic (photo)chemistry have also emerged [44‑50]. XAS can be used both in steady state and in time‐resolved modes [33, 35‑38] and provides valuable information that is complementary to that obtained with the more common UV‐visible and infrared spectroscopies.

Due to the large differences between the core binding energies of different elements, a near‐edge X‐ray absorption fine structure (NEXAFS) spectrum of a specific element provides information related to that atom type. For pyridine N‐oxide and pyridazine N‐oxide, this means that it is possible to obtain information about molecular changes involving the carbon, nitrogen, and oxygen atoms independently. In this study we have collected the steady‐state and time‐resolved spectra of pyridine N‐oxide **1** and pyridazine N‐oxide **2** in water at both the nitrogen and oxygen K‐edges. The sample is a liquid sheet flowing at such a rate that the sample is fully refreshed between pump/probe cycles comprising excitation pulses from a femtosecond laser (258 nm) and X‐ray probe pulses from the BESSY synchrotron [33].

The spectral data, in combination with reference spectra and calculations, allow us to track the dynamics of the structural evolution of these two molecules in water. We simultaneously observe the molecular rearrangements along the singlet pathway, as well as the release of atomic oxygen (O(^3^P)) by its characteristic absorption at the oxygen K‐edge.

In the ground state, pyridine N‐oxide **1** has c_2v_ symmetry. Its frontier Molecular Orbitals (MO's) are shown in Figure S2 (Supporting Information). Low‐lying excited configurations arise from promotion of an electron from the highest occupied π (a_2_, b_1_) and n (b_2_) MO's to the lowest unoccupied π*(a_2_, b_1_) MO's, giving rise to a weakly allowed ππ* transition (1A_1_ → 1B_2_), a strongly allowed ππ* transition (1A_1_ → 1A_1_), and a forbidden nπ* (1A_1_ → 1A_2_). Based on UV absorption studies, Ito and Hata assigned the lowest‐energy absorption features in the gas phase (and in nonpolar n‐hexane) to the nπ* transition [51, 52], but later work favors the 1B_2_ ππ* state as the lowest singlet excited state [53‑56]. On the other hand, Palmer et al. report that the nπ* 1A_2_ state is the lowest vertically excited state according to MRD‐CI and time‐dependent density functional (TDDFT) calculations [57]. Our calculations using the B3LYP or CAM‐B3LYP hybrid functionals at the B3LYP/def2‐QZVP geometry give the same result as Palmer et al. [57]. (See Supporting Information). For the present study, using water as the solvent, there is no doubt that the ππ* state is the lowest‐energy excited state, because the n‐orbital is strongly lowered in energy by the hydrogen bonding interaction, which leads to a large blue shift of the nπ* absorption. As we will show below, the excited states S_1_ and T_1_ undergo distortion to a nonplanar structure. From the S_1_ state a minimum energy crossing point (MECP) is reached at which return to the ground state occurs, forming reactant **1**, oxaziridine **3,** and possibly oxazepine **4**.

## 2 Results

### 2.1 Steady‐State NEXAFS of Compounds 1 and 2

**FIGURE 2:**
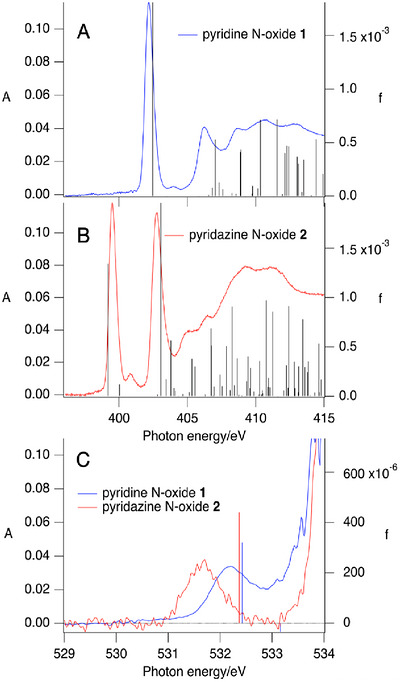
Steady‐state NEXAFS of (A) pyridine N‐oxide **1** and (B) pyridazine N‐oxide **2** in water (0.1 M) at the nitrogen K‐edge and (C) at the oxygen K‐edge. The vertical bars are calculated transitions (section 2.5) with oscillator strengths on the right axis.

The nitrogen K‐edge absorption spectra of **1** and **2** are shown in Figure 2, together with the transitions computed using the transition potential method (see below) [58‑61]. The absorption peak center positions and molar absorption coefficients are given in Table 1.

**TABLE 1:** NEXAFS absorption features of pyridine N‐oxide **1** and pyridazine N‐oxide **2** at the nitrogen and oxygen K‐edges (Figure 2).

*E* (eV) ^[^ ^a^ ^]^	ε (M^−1^µm^−1^) ^[^ ^b^ ^]^	*E* (eV) ^[^ ^a^ ^]^	ε (M^−1^µm^−1^) ^[^ ^b^ ^]^
Pyridine N‐oxide 1		Pyridazine N‐oxide 2	
402.2	0.116	399.5	0.119
404.0	0.005	400.8	0.013
406.2	0.041	402.8	0.112
408.6	0.040	404.9	0.038
410.7	0.045	406.5	0.048
‍412.9	0.043	409.3	0.079
		411.3	0.079
‍		415.3	0.060
532.2	0.037	531.7	0.042

^[a]^ Photon energy.

^[b]^ Molar absorption coefficient.

The nitrogen K‐edge spectrum of pyridine N‐oxide **1** in water contains an intense pre‐edge absorption band centered at 402.2 eV and a broader and less intense feature at 406.2 eV. These features are characteristic of 1s → π^∗^ and 1s → σ^∗^ transitions [34], as supported by the quantum chemical calculations discussed in section 2.5. The first absorption peak corresponds to the N1s → LUMO transition. An absorption continuum corresponding to the ionization from the N1s core level is present at a few eV higher energy, the energy difference being the electron affinity. The post‐edge features at 408.6 eV and above may be attributed to transitions to high‐energy σ^∗^ and Rydberg states and shape resonances.

The absorption spectrum of pyridazine N‐oxide **2** contains two intense absorption peaks at 399.5 eV and at 402.8 eV. These strong pre‐edge features are consistent with its expected 1s → π^∗^ transitions due to the presence of two chemically different nitrogen atoms in the molecule. The first intense peak at 399.5 eV is found at a similar energy as the N1s → π^∗^ transition of pyridine (399.0 eV) [48], and the second intense peak at 402.8 eV is close in energy to the first intense feature of pyridine N‐oxide **1**. At 404.9 eV and 406.5 eV, a pair of peaks is assigned to the two N1s → σ^∗^ transitions. As with pyridine N‐oxide **1**, features at higher energies are provisionally assigned as transitions from 1s to σ^∗^ and Rydberg orbitals as well as shape resonances.

The available spectral window in the O K‐edge region is limited to energies below 534 eV because of the strong absorption by the solvent above this energy. Within this spectral window we can detect a single broad band at 532.2 eV for pyridine N‐oxide **1** and one at 531.7 eV for pyridazine N‐oxide **2**. Being the lowest energy transition in this range, these bands can safely be assigned as O1s → LUMO transitions. In Figure 2 we also added the quantum chemically predicted spectra, discussed in more detail in section 2.5.

### 2.2 Time‐Resolved NEXAFS

**FIGURE 3:**
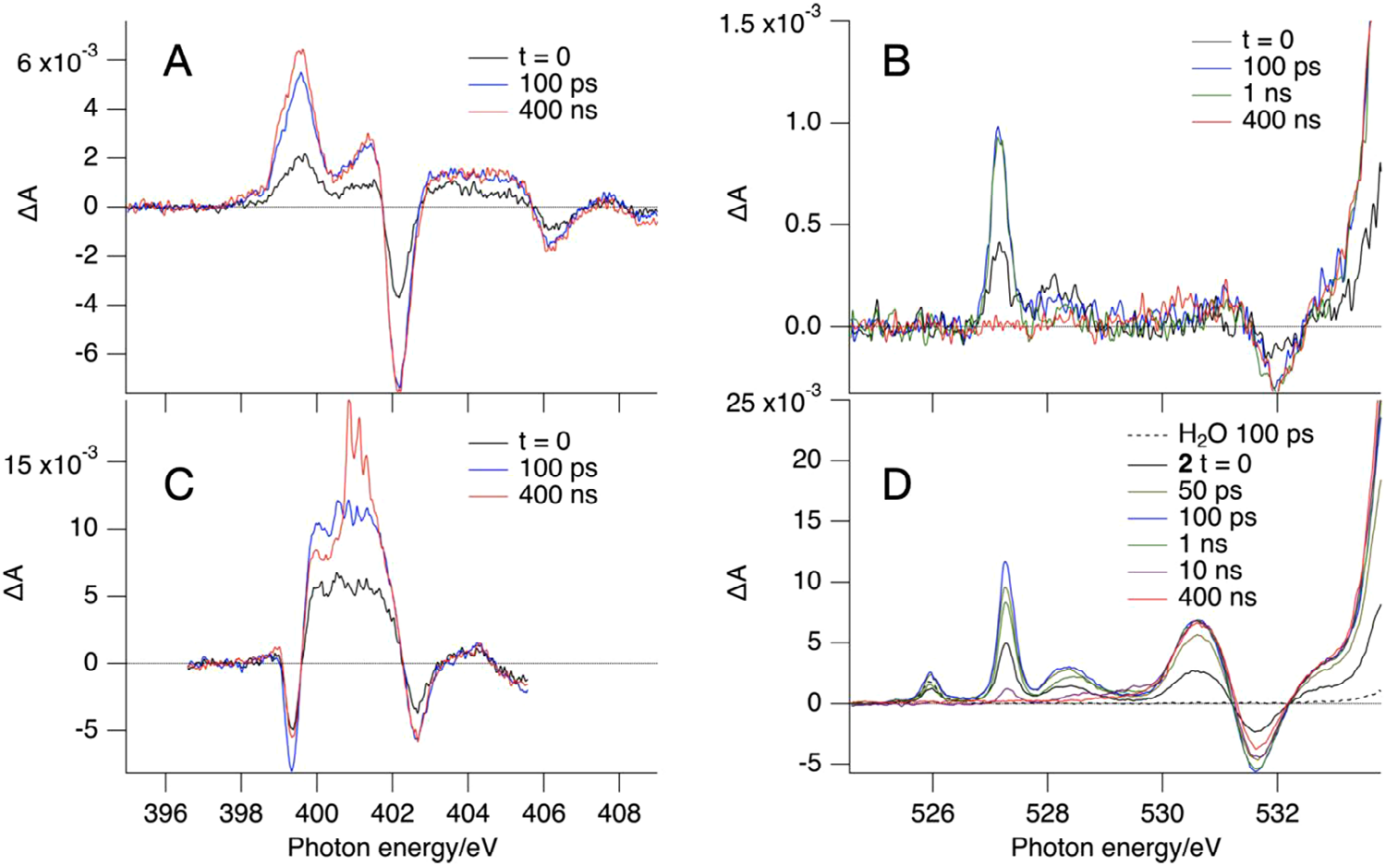
X‐ray transient absorption difference spectra at different times after the 258 nm excitation pulse. (A). Nitrogen K‐edge of pyridine N‐oxide **1**. (B). Oxygen K‐edge of **1**. (C). Nitrogen K‐edge of pyridazine N‐oxide **2**. (D). Oxygen K‐edge of **2**. “t = 0” refers to the maximum of the instrument response function.

The nitrogen and oxygen K‐edge transient absorption spectra of pyridine N‐oxide **1** and pyridazine N‐oxide **2** are shown in Figure 3. These reveal the difference spectra (UV pump + X‐ray probe vs. probe‐only) following the excitation of the sample by a UV pulse at different delay times between the excitation and the probing X‐ray pulse. In the oxygen K‐edge spectra we have included the transient absorption signal of pure water at a 100 ps delay for comparison, measured immediately after under the same conditions for each of the experiments.

**FIGURE 4:**
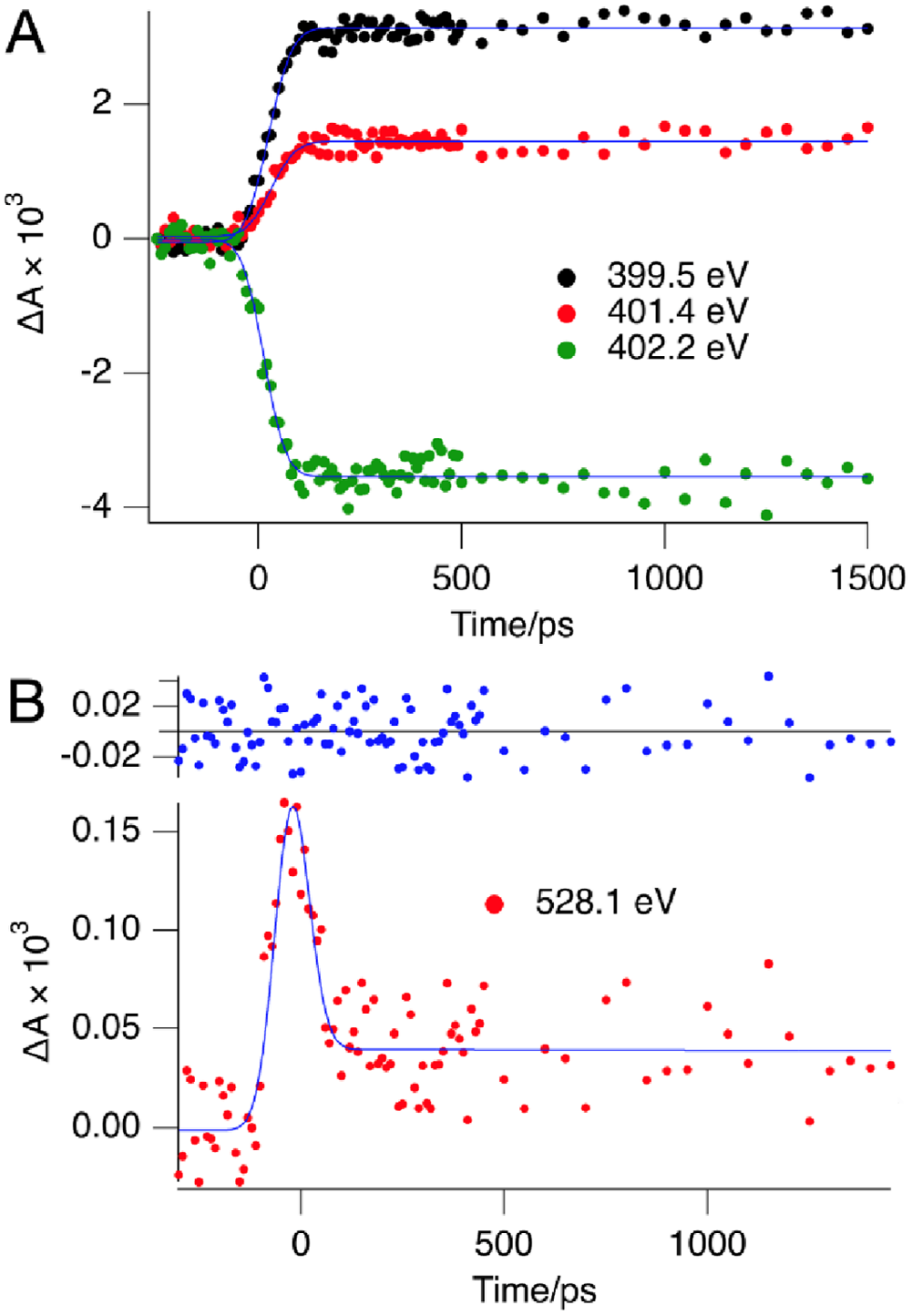
X‐ray transient absorption time traces of pyridine N‐oxide **1** following pulsed excitation at 258 nm. Data are shown as dots fits as blue lines. Time traces were measured at the most intense features in Figure 3A. (A). Nitrogen K‐edge traces fitted to the integral of a Gaussian instrument response function. B. Time trace at 528.1 eV (oxygen K‐edge) fitted to a biexponential decay convolved with a Gaussian function. The blue markers are the residuals.

For the nitrogen K‐edge of pyridine N‐oxide **1,** we collected the time trace of the ground state bleach at 402.2 eV and of two transient absorption features at 399.5 eV and 401.4 eV (Figure 4). Since the absorption does not change in the time window of 1.5 ns after reaching a plateau value, we fitted the curves to a cumulative distribution function, which is the integral of the Gaussian instrument response function (IRF). A Gaussian IRF is commonly assumed in transient absorption spectroscopy [62‑65], and the good fits in Figure 4A support its use. The full width at half maximum of the IRF is ∼100 ps, slightly longer than the time response of the setup reported earlier [33]. The small feature at 528.1 eV decays almost within the response time (fitted lifetime ∼10 ps). It may be due to the excited state absorption of pyridine N‐oxide **1**. The spectra (Figure 3A, B) show no further evolution up to 400 ns, indicating that the primary photochemical reactions of pyridine N‐oxide **1** are completed within 100 ps. Interpretation of the spectral data is discussed below.

**FIGURE 5:**
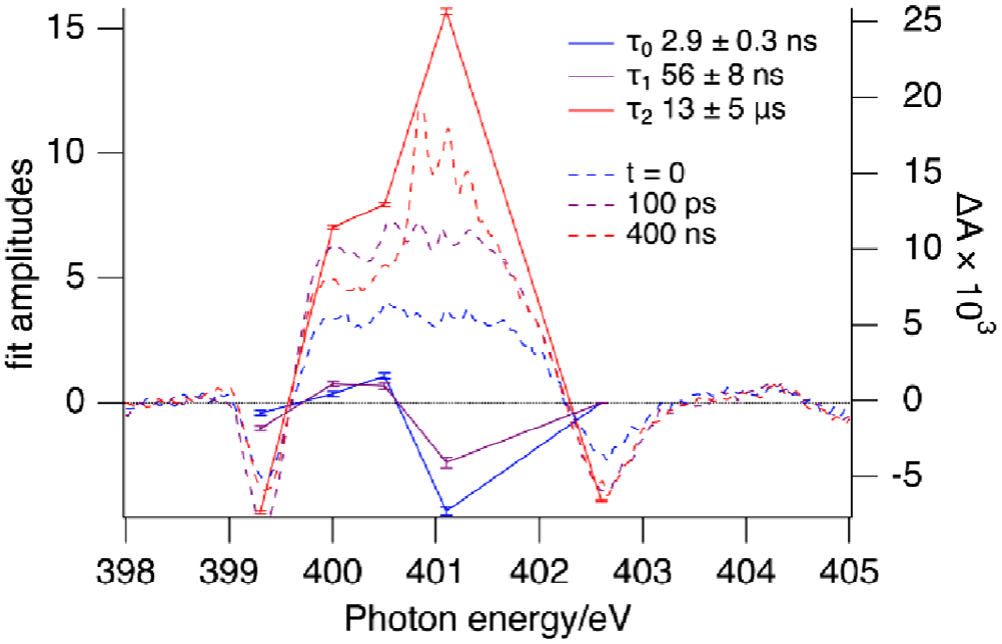
Comparison of the amplitudes (solid lines (blue A_0_, purple A_1_, red A_2_)) of the global fits of the time traces of pyridazine N‐oxide **2** at the nitrogen K‐edge (Figure 5 and Table 2) with the transient spectra at different times (dashed lines) (same as in Figure 3C).

**FIGURE 6:**
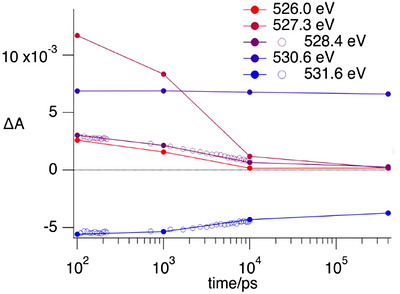
Cross sections of the transient absorption spectra of pyridazine N‐oxide **2** at the oxygen K‐edge (Figure 3D) along the time axis (filled circles), combined with the time traces (Figure S4) at 528.4 and 531.6 eV (open circles).

The time traces collected for pyridazine N‐oxide **2** are shown in Figure S4. The fitted time constants and amplitude values can be found in Table 2. The traces at 399.3 eV and 402.6 eV track the ground state bleach, while those at 400.0 eV, 400.5 eV, and 401.1 eV follow transient absorption features. The traces at the nitrogen K‐edge and at the oxygen K‐edge could be globally fitted with three lifetimes of 3 ns, 56 ns, and 13 µs (Table 2) [62‑65]. In Figure 5, the amplitudes associated with the three temporal components are shown together with the experimental spectra at three delay times. At short delay times (Figure S4A), the traces show the features rising within the temporal resolution of the experiment. The ground state bleach band (GSB) at 402.6 eV shows only a very slow recovery. The fitted time constant of 13 µs for this slow recovery is not accurately determined in the available time window of 400 ns. The other GSB band, at 399.3 eV, shows an additional rise with the 56 ns component, suggesting that a product is formed that has an absorption at this photon energy. The photoinduced absorption features at 400.0 eV and 400.5 eV show a small decrease in their signals, with minor contributions from the nanosecond components. The absorption at 401.1 eV increases over time, up to ∼200 ns, and then starts to slowly decay. Figure 5 clearly illustrates that the rise of the structured feature at ∼401 eV is accompanied by decreased absorption at ∼400 eV. At the oxygen K‐edge we observe a decrease of the amplitude of the GSB at 531.6 eV with a lifetime of 3 ns and a second slower component of 56 ns. The excited state absorption band at 528.4 eV shows a decay with both time constants. Combined time profiles from Figure 3D and FIgure S4 are shown in Figure 6.

**TABLE 2:** Fits of the single‐energy time traces of pyridazine N‐oxide **2**. The curves were fitted globally with a sum of up to three exponentials convolved with a Gaussian IRF. The FWHM of the Gaussian was found to be ∼75 ps. The three lifetimes recovered are τ_i_, associated amplitudes at different energies are A_i_.

E (eV)	A_0_ (× 10^3^)^[^ ^a^ ^]^	A_1_ (× 10^3^)^[^ ^b^ ^]^	A_2_ (× 10^3^)^[^ ^c^ ^]^
399.3	−0.4 ± 0.10	−1.02 ± 0.10	−4.37 ± 0.06
400.0	0.37 ± 0.10	0.76 ± 0.11	7.05 ± 0.07
400.5	1.09 ± 0.11	0.69 ± 0.12	7.96 ± 0.07
401.1	−4.33 ± 0.15	−2.37 ± 0.21	15.71 ± 0.12
402.6			−3.92 ± 0.04
528.4	2.3 ± 0.1	1.1 ± 0.1	
531.6	−0.3 ± 0.1	−5.6 ± 0.1	

^[a]^ τ_0_ = 2.9 ± 0.3 ns,.

^[b]^ τ_1_ = 56 ± 8 ns,.

^[c]^ τ_2_ = 13 ± 5 µs.

### 2.3 Computation of the Ground State Reactants and Products

**FIGURE 7:**
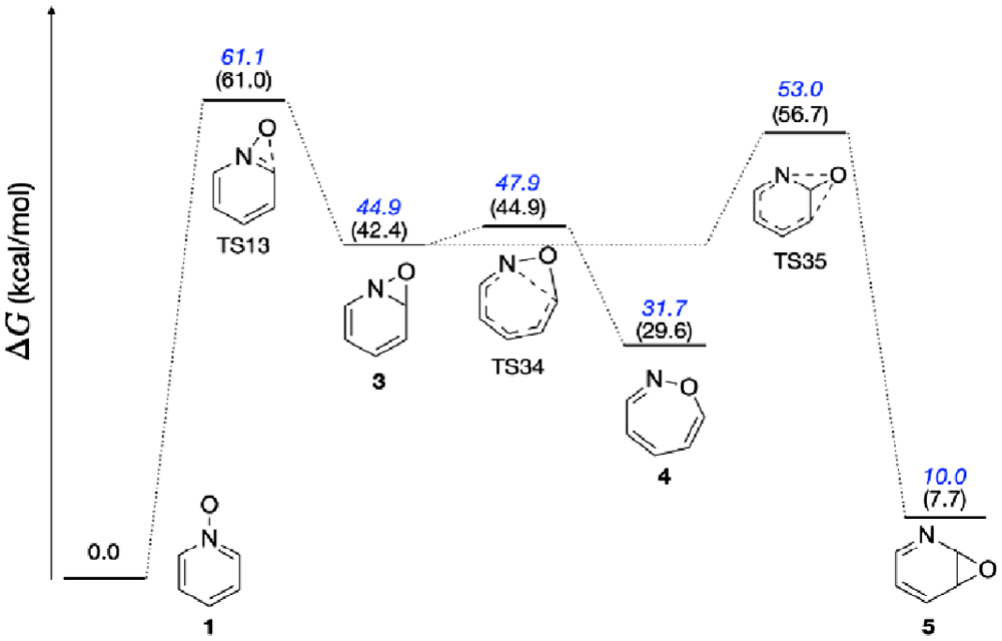
Relative vibrationally corrected energies at 298.15 K for pyridine N‐oxide **1,** its isomers **2**, **3**, and **4**, and transition structures linking the oxaziridine **3** to the other isomers. Ground state, B3LYP/def2‐QZVP. Energy values in black (in parentheses): isolated molecules; values in blue: using the polarizable continuum model for water.

The structures and energies of pyridine N‐oxide **1** and its potential products were computed using the hybrid B3LYP functional [66, 67] with the def2‐QZVP basis set [68, 69]. To account for the solvent effect, the polarizable continuum model for water was employed. Specific interactions of pyridine N‐oxide **1** with water molecules were also considered (see Supporting Information). Figure 7 schematically shows the relative energies (corrected for thermal contributions) of minima and transition structures for pyridine N‐oxide **1** and its isomers. Table S1 lists all energies. Molecular structures are available in the Supporting Information.

For the case of pyridazine N‐oxide **2**, Ma et al [32] studied the energy minima and transition structures at various levels of theory. In the present work, we have applied the B3LYP/def2‐QZVP approach throughout. An energy diagram for pyridazine N‐oxide **2** and its isomers analogous to Figure 7 is provided in the Supporting Information (Figure S1 and Table S2). We find oxaziridine **7** to be a local minimum on the potential energy surface when using the PCM solvation model, with the transition structure for 6π electrocyclic ring opening only 0.04 kcal/mol higher in energy, but the barrier vanishes when thermochemical corrections are applied. Experimentally, oxaziridine **7** has been shown to be the primary photoproduct of pyridazine N‐oxide **2**, and its long lifetime demonstrates that the computed barrier has a surprisingly large error [32]. In acetonitrile oxaziridine **7** transforms on a time scale of 900 ns into the ring‐opened 4‐diazobut‐2‐enal **9,** presumably via the oxadiazepine **8**, which is not observed experimentally and was also not found as an energy minimum in geometry optimization with a variety of computational methods. Ma et al. also identified 1,3,4‐oxadiazepine **11** as a product, which might arise from oxaziridine **10,** which in turn originates from a ring‐walk rearrangement of oxaziridine **7**. We located a shallow local minimum for **10** but failed to find a transition structure for the ring walk **7** to **10**. The transition structure for ring opening of **7** leads directly to **9** (cZc conformer), which rearranges to a mixture of conformers of which cZt is the most stable (ΔΔG_0_ = 3.6 kcal/mol in water).

### 2.4 Computation of the Excited State Dynamics

The computational description of the excited‐state dynamics of aromatic N‐oxides presents a diverse set of challenges. The dynamics may involve the crossing from a singlet to a triplet surface, as well as the crossing from an excited state to the ground state via a conical intersection or a MECP. The MECP is the point with the lowest energy at which the excited and ground state energies are degenerate. The optimization of the excited state and location of the MECP of **1** and **2** was performed using state‐averaged complete active space self‐consistent field theory with MP2 correction (SA(4)‐CASPT2) using an ANO‐RCC‐VDZP basis [70].

To gain some further insight into the electronic structure of pyridine N‐oxide **1**, TDDFT methods were used. Vertical excitation at the ground state optimized geometry using B3LYP or CAM‐B3LYP (def2‐QZVP basis) predicted the nπ* (A_2_) state to be the lowest excited singlet state in the gas phase and nonpolar solvents, but when using the PCM model for water, the B_2_ ππ* was the lowest excited singlet state. Including explicit water molecules, hydrogen‐bonded to the electron‐rich oxygen atom, drastically raised the nπ* energy level due to the lowering of the energy of the lone pair on oxygen.

**FIGURE 8:**
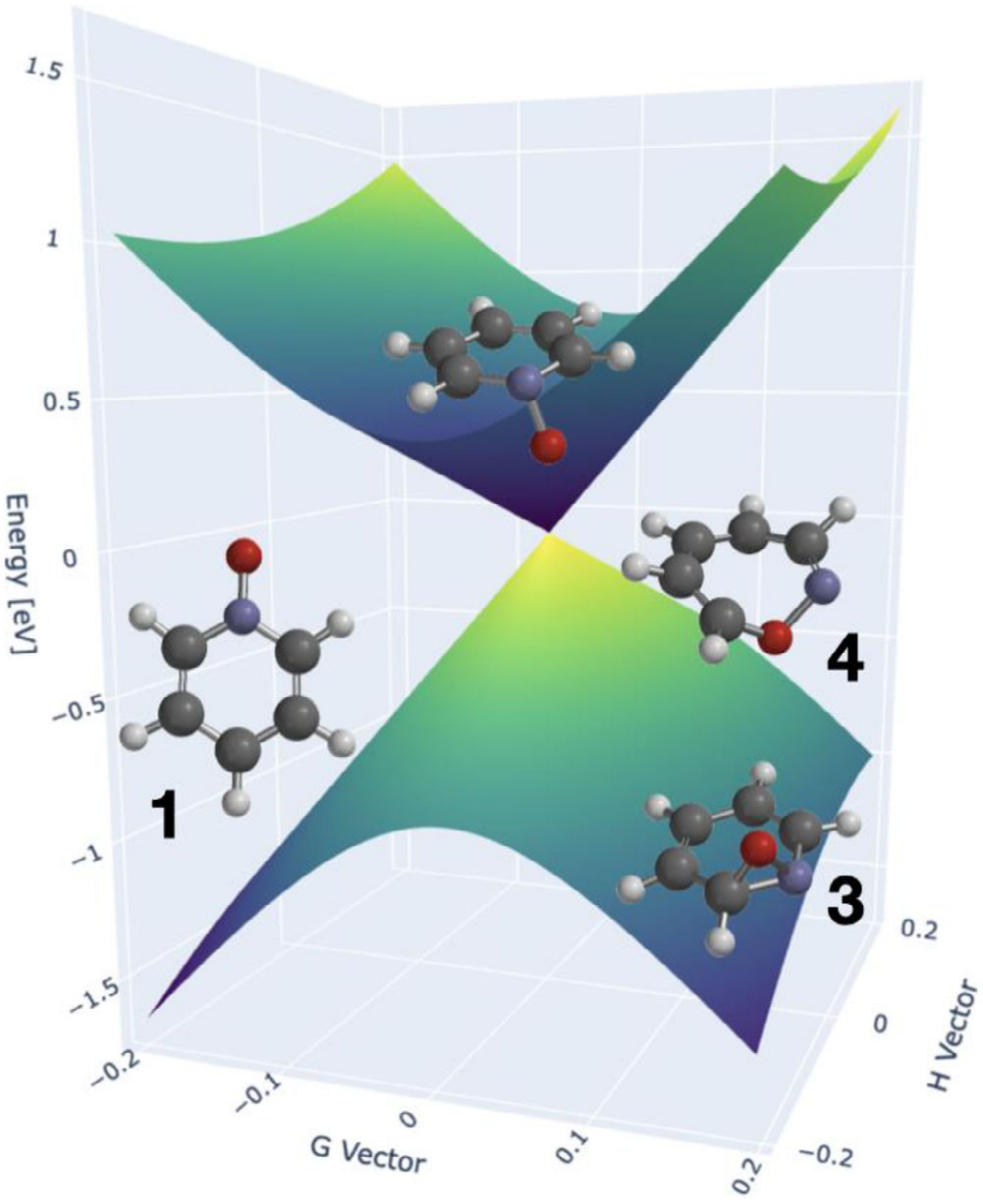
Graphical presentation of the energy surfaces of pyridine N‐oxide **1** around the minimum energy crossing point (MECP) (SA(4)‐CASPT2/ ANO‐RCC‐VDZP) and the products **1**, **3**, and **4** that can be reached directly from it [72].

Geometry optimization of the S_1_ state with TDDFT and of the T_1_ state with UB3LYP led to displacement of the oxygen atom out of the plane of the aromatic ring, resulting in symmetry lowering from c_2v_ to c_s_. For the isolated molecule, c_s_ minima were found both in S_1_ and T_1_. When using PCM water, further symmetry lowering of S_1_ occurred, leading to structures with an energy gap of less than 0.1 eV with the ground state. Full optimization near a conical intersection cannot be achieved with TDDFT, but the CASPT2 calculation found the MECP with a structure very similar to the lowest‐energy one found with TDDFT, shown in Figure 8. A transition state connecting the FC S_1_ structure and the MECP could not be located.

The spin‐orbit coupling element V_SO_ was computed both at the FC S_1_ and the MECP geometries using the RASSI method. This element was found to be V_SO_ = 0 at the FC geometry, but V_SO_ = 10 cm^−1^ at the MECP geometry. This suggests that the rate of intersystem crossing will be appreciable near the MECP geometry, where the S_0_, S_1_, and T_1_ geometries become isoenergetic. The picture that emerges from both TDDFT and CASPT2 calculations is one of an ultra‐fast barrierless geometry distortion from the excited pyridine N‐oxide toward the MECP, which serves as a branching point from which the molecular trajectory can reach the T_1_ and S_0_ surfaces. From the MECP structure, the system evolves on the ground state surface in a branching plane spanned by the gradient difference $\vec{g}$ and the derivative coupling $\vec{h}$ vectors [71‑73]. Moving along $\vec{g}$, **1** and oxaziridine **3** are formed. By moving along $\vec{h}$, or a combination of $\vec{g}$ and $\vec{h}$, recoupling of the unpaired electrons can lead to oxazepine **4** directly, bypassing the oxaziridine **3**, as illustrated in Figure 8.

These observations help to generate hypotheses about the ground state evolution and product formation, but for making meaningful quantitative computational predictions, more sophisticated methods are required, beyond the scope of the present study. A solvent model will be necessary to address the experimentally observed differences in the photochemistry of pyridazine N‐oxide **2** in acetonitrile (ref. [32]) and water (this work). The difficulty in correctly predicting the barriers on the ground state surface poses another challenge.

### 2.5 Computation of XAS Spectra

For the interpretation of the experimental data, the computed XAS of reactants and products are very useful. A resource‐efficient and fairly accurate method for computing the X‐ray absorption spectra of molecules is the transition‐potential density functional theory approach [58‑60]. The limited benchmarks of this method available show that using the B3LYP functional with the def2‐QZVP basis produces spectra that match the near‐edge X‐ray absorption well. To account for a small systematic deviation between computed and experimental spectra [60, 61], we shifted the computed N‐edge spectra by ‐2.18 eV, which is the average offset of the experimental vs. calculated peak positions for the N1s to LUMO transitions in pyridine [48], pyridine N‐oxide **1**, and pyridazine N‐oxide **2**. For the O K‐edge, the data from pyridine N‐oxide **1**, pyridazine N‐oxide **2**, and the two oxygen atoms of thymine [59] were used, giving a correction of ‐0.24 eV.

**FIGURE 9:**
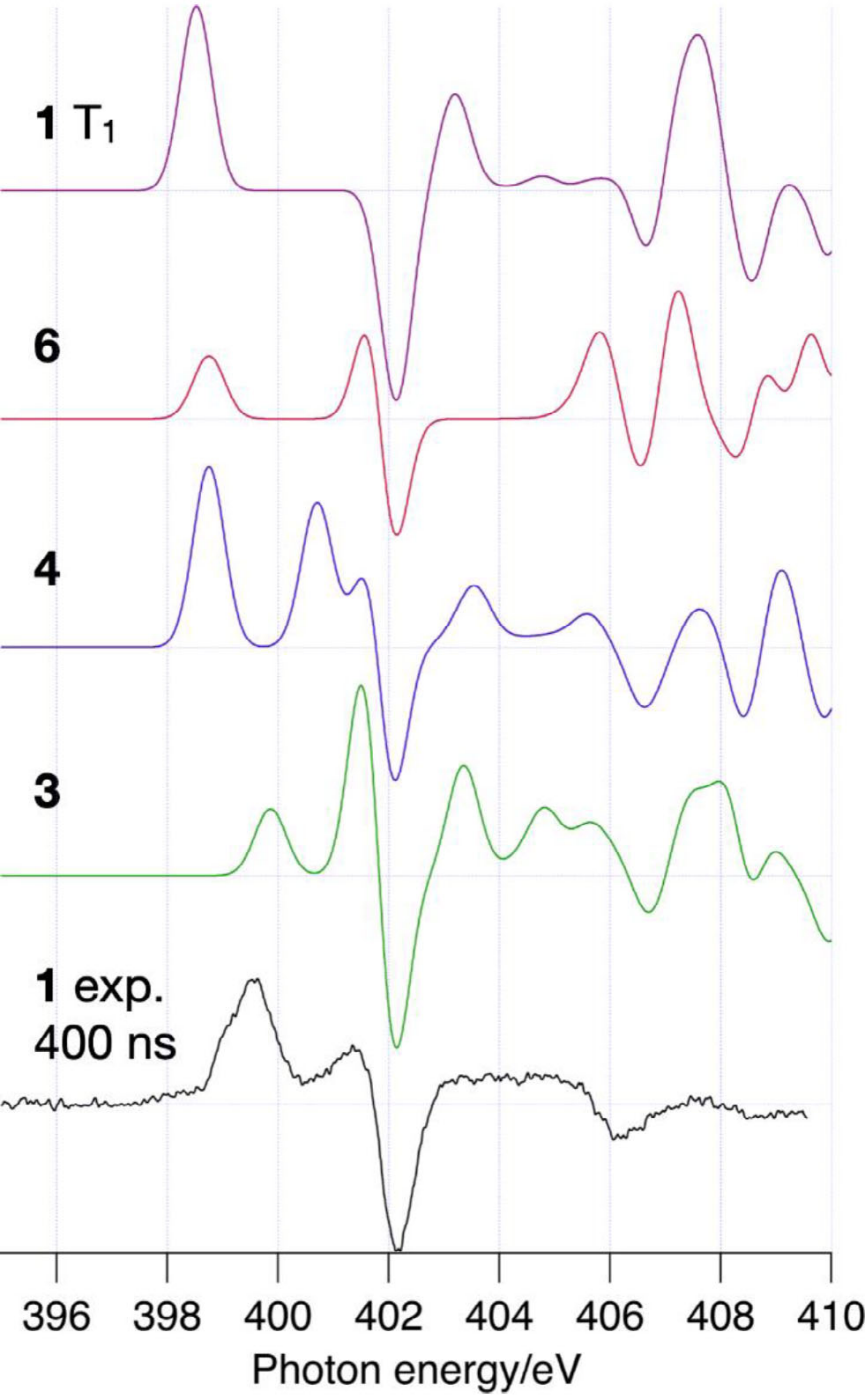
Simulated XAS difference spectra (nitrogen K‐edge) of the candidate products compared with the experimental spectrum of **1** at 400 ns (Figure 3A).

**FIGURE 10:**
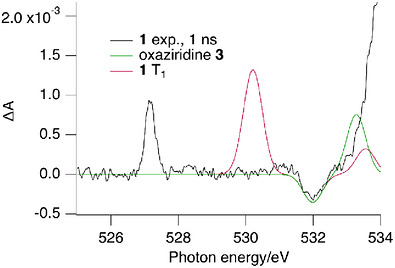
Simulated XAS difference spectra (oxygen K‐edge) of the candidate products compared with the experimental spectrum of **1** at 1 ns (Figure 3B). The calculated peaks of oxazepine **4** are at >535 eV, hidden by the solvent absorption.

Candidate photoproducts that may contribute to the time‐resolved XAS of pyridine N‐oxide **1** (Figure 3A) are oxaziridine **3**, oxazepine **4**, the triplet state of pyridine N‐oxide, and pyridine **6**. Figure 9 shows the calculated difference spectra of the potential products with that of the ground state of **1**, shifted slightly to match the GSB peak at 402.15 eV, together with the experimental spectrum (from Figure 3A). Figure 10 presents the calculated difference spectra of the potential product at the oxygen K‐edge, together with the experimental data (from Figure 3B).

**FIGURE 11:**
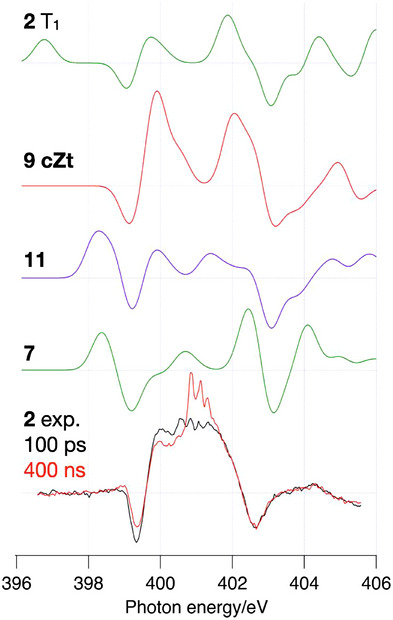
Predicted difference spectra at the nitrogen K‐edge for previously proposed photoproducts of pyridazine N‐oxide **2** compared with experimental difference spectra at 100 ps and 400 ns (from Figure 3C). All computed spectra are scaled according to the predicted oscillator strengths.

**FIGURE 12:**
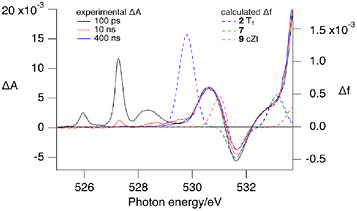
Experimental (Figure 3D) and simulated XAS spectra of **2** at the oxygen K‐edge.

For pyridazine N‐oxide **2**, multiple photoproducts may be expected based on the study of Ma et al. [32]. The oxaziridine **7** in acetonitrile undergoes ring opening to produce (*Z*)‐4‐diazobut‐2‐enal **9**. In addition, the oxadiazepine **11** has been detected in acetonitrile. In Figures 11, 12 we show the predicted difference spectra of **7**, **11**, **9** (in the lowest‐energy conformation), and the T_1_ state of **2** with the spectrum of **2** (subtracted). Additional spectra are shown in the supporting information (Figure S3).

## 3 Discussion

### 3.1 Pyridine N‐oxide 1

Upon excitation of pyridine N‐oxide **1,** we observe the rise of a transient absorption spectrum within the time resolution of the experiment (Figures 3, 4). The spectrum does not change between 100 ps and 400 ns. Bleaching of the ground state absorption is seen at 402.2 eV, 406.2 eV, and at 532 eV. Product signals comprise peaks at 399.5 eV, 401.4 eV and a broad feature between 403 eV and 406 eV. At the oxygen K‐edge a short‐lived feature is detected that might be due to the singlet excited state, other product peaks may be hidden by the strong solvent absorption above 534 eV.

Inspection of Figures 9,  10 shows that oxaziridine **3** is the most likely product. The pattern of the peaks matches the experimental spectrum at the nitrogen K‐edge, and at the oxygen K‐edge the predicted absorption may correspond to the shoulder on the solvent band. The triplet state of **1** is not present: it would have given a prominent band at both edges at lower energy than the GSB band. Pyridine **6** has a large splitting between the first two peaks and lacks the broad transient absorption observed between 402 and 406 eV. Experimentally, the absorption peaks of pyridine are found at 399.0 and 402.8 eV, which implies that pyridine cannot account for the positive feature at 401.4 eV in the transient spectrum. Oxazepine **4** has its first predicted absorption peaks at the nitrogen K‐edge at slightly lower energy than oxaziridine **3**, and the pattern of positive and negative ΔA in the range 406 – 410 eV does not match the experimental spectrum. The absence of a change in the spectra in the 400 ns time window shows that the conversion of **3** to **4** is slow. We cannot exclude, however, that a small amount of **4** is formed directly from the MECP, as predicted theoretically: it might be responsible for the shoulder at the low‐energy edge of the absorption spectrum.

Energies of reactant **1** and its potential primary products are shown in Figure 7 (and Table S1). The computed barrier for the 6π electrocyclic ring opening of oxaziridine **3** (7‐oxa‐1‐azabicyclo[4.1.0]hepta‐2,4‐diene) to 1,2‐oxazepine **4** is only 2.9 kcal/mol, from which we would expect a lifetime of <100 ps, but experimentally it is found to be of the order of hundreds of nanoseconds, or more. This implies that the barrier is underestimated by more than 5 kcal/mol. The same has been reported for oxaziridine **7** in acetonitrile [32]. Epoxide **5**, suggested to be an intermediate in the photochemistry leading to 3‐hydroxypyridine [13], has the lowest energy of the products considered here, but we have no experimental evidence for its formation in the experimental time window. The barrier for the ring walk of **3** to **5** is calculated to be 8.1 kcal/mol, corresponding to a time scale of ∼1 microsecond. An important difference between our experiments and those of Cai et al. is the absence of acid in our case, which likely catalyzes the rearrangement [13]. Based on the calculated XAS spectra, the formation of the triplet state of **1** can be excluded as the primary pathway, and pyridine is not formed to a measurable extent. Also 2‐pyridone (400.7 eV) [48] (the stable tautomer of 2‐hydroxypyridine in water) and 3‐hydroxypyridine (399.1 eV) [48] do not appear to be formed in significant amounts.

Previous work has established that pyridine N‐oxide **1** in basic water yields the open‐chain 5‐hydroxypentadienenitrile with a time constant of about 63 milliseconds [74]. Like pyrrolecarbaldehyde, a product observed in organic solvents [5], this results from two sequential ring‐opening steps. On the time scale of the present experiments, we do not detect these processes.

The spectra at the oxygen K‐edge (Figure 3B) show a transient feature at 527.3 eV, which is detected at t ≈ 0 (within the instrument response function of ∼100 ps) and at 100 ps, but not at later times. This feature is common to the photochemistry of **1** and **2** and will be discussed further below. An excited state absorption at 528.1 eV, barely visible in Figure 3B, was monitored in a time‐resolved scan and decays with a lifetime smaller than the system response width (Figure 4B). This is tentatively attributed to the singlet excited state of pyridine N‐oxide **1**.

### 3.2 Pyridazine N‐oxide 2

The transient spectra of pyridazine N‐oxide **2** (Figures 3C,  D) are richer than those of pyridine N‐oxide **1**. Ground state bleach signals are apparent at 399.3 and 402.6 eV at the nitrogen K‐edge and at 531.6 eV at the oxygen K‐edge. At the nitrogen K‐edge a broad band in the range 400–402 eV rises rapidly, and so do several peaks in the oxygen K‐edge range. On a timescale of nanoseconds, the spectra evolve with reduction of the amplitude of the bleaching and formation of a structured feature near 401.1 eV.

Ma et al. have studied the dynamics of pyridazine N‐oxide **2** upon excitation using transient electronic and vibrational spectroscopies [32], and provided strong evidence for the involvement of oxaziridine **7** and ring‐opened product **9**. The intermediate oxadiazepine **8** was not detected, in agreement with computational data indicating that the oxadiazepine **8** does not have a stable energy minimum. In acetonitrile the oxaziridine **7** is formed with a time constant of 11 ps [32] and, if the reaction is similarly fast in water, bands due to **7** should appear in the first transient spectrum (Figure 3C). Looking at Figure 12, however, we see no evidence for the presence of oxaziridine **7** in the transient spectra: the expected low‐energy absorption band at the nitrogen K‐edge is absent. Oxadiazepine **11** does not appear to be present either. We confirmed that oxaziridine **7** is predicted to have its first nitrogen K‐edge absorption at a lower energy than pyridazine N‐oxide **2** at the ΔSCF level of theory, the most accurate among the computational methods explored by Jana and Herbert (Table S5) [61]. Using this method, the absorption is predicted at an even lower energy, so if oxaziridine **7** would be the primary product, it should have been observed at the nitrogen K‐edge. Both the nitrogen K‐edge and the oxygen K‐edge confirm the presence of the diazo product **9**. Since **9** lives much longer than the time window of our experiment, the fitted 13 µs is only a rough indication of the actual lifetime.

Interestingly, the experimental nitrogen K‐edge spectrum at 100 ps shows that the vibrational features characteristic of the diazo group [32] are already present at this time. The same is true for the absorption at 530.6 eV at the oxygen K‐edge, which can also be attributed to diazo compound **9** based on the TP calculations. A tentative explanation for the rapid formation of **9** is that decay from the MECP in the case of **2** leads mostly toward 1, 2, 3‐oxadiazepine **8**, which is not an energy minimum and decays to **9**. The product is formed in the less stable cZc conformation, and the dynamics with time constants of ∼3 ns and ∼56 ns can be attributed to conformational relaxation of **9**. Incidentally, the XAS spectrum of N_2_ [75, 76] resembles that of the final product of pyridazine N‐oxide. N_2_ can indeed be formed by decomposition of the diazo compound **9** [77, 78], but it seems unlikely that this would occur on a time scale of 56 ns [32, 79]. Moreover, the formation of **9** from pyridazine N‐oxide **2** is well established, the diazo unit can be expected to give rise to a similar vibrational progression (2080 cm^−1^) [32] in its spectrum as N_2_, and the TP calculations support the detection of **9** at the oxygen K‐edge [32]. A time‐resolved IR measurement of pyridazine N‐oxide **2** in water would give conclusive evidence.

Like in the case of pyridine N‐oxide **1**, TP‐DFT calculations predict that at the oxygen K‐edge, the closed‐ring intermediates absorb at higher energies, so they are hidden by the solvent absorption.

In Figure 3D, we observe additional features that can be attributed to the formation of the O(^3^P) atom and to the hydroxyl radical, as discussed in the next section [80‑82]. If deoxygenation occurs, as in pyridine N‐oxide, pyridazine should be a product as well. Unfortunately, its absorption peaks overlap strongly with those of the starting compound [83].

### 3.3 Reactive Oxygen Species

The lowest‐energy transient absorption observed at the oxygen K‐edge at 526 eV can be assigned to the OH radical [80‑82]. It is formed in the absence of the N‐oxides as well due to the two‐photon ionization of water [84]. A peak at 527.3 eV appears both in the spectrum of pyridine N‐oxide **1** (Figure 3B) and that of pyridazine N‐oxide **2** (Figure 3D). In the latter case all signals are stronger, possibly due to a better optimized pump‐probe spatial overlap or higher pump fluence, and the temporal evolution could be tracked in more detail, showing that the signal decays in 10 ns (Figure 6). The absorption of O(^3^P) has been measured to be at 526.8 eV in the gas phase [85, 86] and 527.4 eV in a solid ice matrix [80], so we assign the signal at 527.3 eV to O(^3^P), a well‐known potential photoproduct during the photolysis of aromatic N‐oxides.

It has been claimed that the triplet state is the precursor of oxygen atoms in the photolysis of **1** [20, 87], but in 4‐benzoylpyridine N‐oxide, the triplet was spectroscopically detected, and it was shown not to be the source of the oxygen atoms [21]. In the present cases, we see an instantaneous rise of the absorption of the oxygen atom, and we have no evidence for the existence of the triplet state of the N‐oxide on a longer timescale. Thus, either fast inter‐system crossing produces a very short‐lived triplet, or the oxygen atoms arise directly from the singlet state. Together with oxygen atoms, the formation of pyridine and pyridazine is expected, but we do not have evidence for these. Spectral overlap with other species [83] and relatively low yields may have been the cause of this. The band at 528.4 eV in the transient absorption spectrum of pyridazine N‐oxide **2** remains so far unassigned. It cannot be due to one of the ground state photoproducts. Maybe it originates from a complex of pyridazine with the oxygen atom, recombining on a < 10 ns time scale to pyridazine N‐oxide **2** or a hydroxypyridazine.

## 4 Conclusion

When considering all data, the following picture arises: on a very short timescale, the excited N‐oxides **1** and **2** reach a MECP, from where they decay on the ground state surface, re‐forming the starting compound, or move toward the oxaziridine and ox(di)azepine isomers. Also, O(^3^P) atoms are formed, which are short‐lived and probably recombine with the aromatic ring to re‐form the starting compounds. In the case of pyridine N‐oxide, oxaziridine **3** is identified as the main product, with a lifetime exceeding the 400 ns observation time window. Oxazepine **4** may be formed as a minor primary photoproduct but not via the thermal ring opening of **3**. In the case of pyridazine N‐oxide, the main product diazobutenal **9,** is formed rapidly (<100 ps), and intermediates are not detected. This is different from a previous study that used organic solvents, in which oxaziridine **7** is the primary photoproduct of **2**, undergoing ring opening to **9** on a timescale of 900 ns in acetonitrile [32]. The computed barrier toward ring opening of **3** is substantially higher than that of **7**, in agreement with the observation that **3** (in water) is long‐lived. Both barriers are seriously underestimated, however, which offers an interesting challenge for computational chemistry.

The present work demonstrates that soft X‐ray probing in transient absorption spectroscopy opens new opportunities for organic photochemistry. There is plenty of room for technological developments that can make the experiments much more powerful in the near future, for example, by using multi‐wavelength detection, as is the standard in visible and infrared time‐resolved absorption spectroscopy, and by the development of table‐top pulsed soft X‐ray sources [37, 47, 88], which can relieve the access restrictions inherent to synchrotron and free electron installations and provide femtosecond time resolution. The computational prediction of X‐ray absorption spectra is a lively area of current research. More robust methods that not only reduce the mean errors but also avoid large individual errors will be an essential companion for the experimental studies.

## 5 Experimental and Computational Details

UV/Vis absorption measurements were performed using a Shimadzu UV‐2700 spectrophotometer. The static and time‐resolved XAS experiments were performed at the BESSY II beamline UE52‐SGM [89], using an experimental setup that has been previously described in detail [33].

The samples were dissolved in ultrapure water at a concentration of 100 mM. An HPLC pump was used to flow the samples into a pair of nozzles enclosed within a vacuum chamber. A flow rate of 1.4 mL/min was used. The two liquid jets that are ejected from the nozzles are aligned such that they collide, producing a stable leaf‐shaped disk with a thickness of 1 ‐ 10 µm. An N_2_‐cooled trap captures the liquid at the bottom of the chamber. Having such a thin sample allows us to measure the transmitted X‐rays, while the relatively high concentrations allow us to excite a large number of molecules with the pump beam. The actual thickness in our experiments was determined to be approximately 6 µm by measuring the absorption through the pure water jet. If we approximate the width of the jet to be 1 mm, then the volume of the horizontal slit that contains the probed region is about 0.48 nL, so with a 1.4 mL/min per minute flow rate, the sample will be refreshed in between the hybrid bunch pulses that arrive every 800 ns. Energy calibrations at the nitrogen K‐edge were performed by measuring the difference NEXAFS spectrum of a sample of iron(II)‐trisbipyridine in water at a delay of 100 ps after the pump and using the absorption feature at 399 eV [33]. The calibration of the O K‐edge region was performed using the transient signal observed in pure water at a 100 ps pump/probe delay. At this delay there is a weak but observable feature in the lower energy end of the spectrum that we attribute to the hydroxide radical that is known to form from the ultrafast decay of the H_2_O^+^ radical cation formed due to two‐photon absorption. The hydroxyl radical has been reported to absorb at 525.93 eV [82, 84]. The energy axis was shifted to match this feature.

The laser source was a commercial 30 W fs‐laser system from Amplitude, which produced 300 fs pulses at the fundamental wavelength of 1030 nm. The pump pulses were frequency‐doubled twice to produce 258 nm pulses at a repetition rate of 208 kHz and a power of 6 µJ per pulse. The pump pulses were focused at the sample to a spot with an FWHM of 80 µm. The time resolution is determined by the X‐ray pulse [33].

Density functional geometry optimizations and frequency calculations were performed using Gaussian 16 [90]. The N and O K‐edge absorption spectra were calculated using the Transition Potential Density Functional Theory (TP‐DFT) using the Psi4 program [59, 60]. The ΔSCF calculations of 1s—LUMO transitions were performed using Q‐Chem 6.1 [91]. CASPT2 calculations were performed using OpenMolcas [72, 73, 92]. For analysis of the time‐resolved data, Igor Pro v.8 was used [93].

## Supporting Information

Supporting information comprises a pdf file containing tables with the energies of all computed structures, an energy diagram of the ground state isomers of **2**, additional computational results on the excited states of **1** (including the effect of explicit water molecules), and additional XAS calculations, and a plain text file with the coordinates of relevant structures in xyz format.

The authors have cited additional references within the Supporting Information [94].

## Conflicts of Interest

The authors declare no conflicts of interest.

## Supporting information




**Supporting file 1**: chem70483‐sup‐0001‐SuppMat.pdf
