# Supplementary material for: Photochemistry of Aromatic N‐Oxides in Water Probed by Time‐Resolved X‐ray Absorption Spectroscopy
Source: Chemistry. 2025 Nov 29;32(29):e02775. doi: 10.1002/chem.202502775 (PMC13451558; doi:10.1002/chem.202502775)
Supplement: Supplementary file 1 [file CHEM-32-e02775-s001.pdf]

SUPPORTING INFORMATION

Photochemistry of Aromatic N-oxides in Water Probed by Time-Resolved X-ray Absorption Spectroscopy

Maximilian Paradiz Domínguez,† Robby Büchner,‡ Mattis Fondell,‡ and Albert M. Brouwer*†

† Van 't Hoff Institute for Molecular Sciences, University of Amsterdam, Amsterdam, The Netherlands

‡ Institute for Methods and Instrumentation for Synchrotron Radiation Research, Helmholtz-Zentrum Berlin für Materialien und Energie, Berlin, Germany

** E-mail: a.m.brouwer@uva.nl*

Contents

Table S1. Calculated energies of **1** and its isomers	S3
Table S2. Calculated energies of **2** and its isomers	S3
Figure S1. Energy diagram for pyridazine N-oxide **2** and its isomers	S4
Figure S2. Frontier Molecular Orbitals of pyridine N-oxide **1**	S4
Table S3. TDDFT excitation energies of **1**	S5
Excited states of **1**	S6
Figure S3. Structure of hydrate of **1**	S6
Table S4. Structures of computed excited state species	S6
Figure S4. XAS time traces of pyridazine N-oxide **2**	S7
Figure S5. Calculated XAS spectra of **2** and its isomers	S8
Table S5. 1s – LUMO excitation energies using the ΔSCF method	S8
References	S9

Table S1. Energies of the structures in Figure 7 in the main text (B3LYP/def2-QZVP).

	Isolated molecules			PCM water		
	E^a	G_0^b	ΔG_0^c	E^a	G_0^b	ΔG_0^c
**1**	-323.59931	-323.53456	0.00	-323.61039	-323.54554	0.00
**3**	-323.52943	-323.46704	42.37	-323.53636	-323.47393	44.94
**4**	-323.54913	-323.48744	29.57	-323.55685	-323.49505	31.69
**5**	-323.58586	-323.52228	7.71	-323.59334	-323.52961	10.00
**TS13**	-323.49861	-323.43732	61.02	-323.50952	-323.44811	61.14
**TS34**	-323.52435	-323.46300	44.91	-323.53068	-323.46929	47.85
**TS35**	-323.50471	-323.44425	56.67	-323.52187	-323.46114	52.96

[a] Energy in Hartree. [b] Energy with thermal corrections (298.15 K) in Hartree. [c] Free energy difference in kcal/mol.

Table S2. Energies of pyridazine N-oxide and its isomers (Figure S1)(B3LYP/def2-QZVP).

	Isolated molecules			PCM water		
	E^a	G_0^b	ΔG_0^c	E^a	G_0^b	ΔG_0^c
**2**	-339.62171	-339.56971	0.00	-339.63516	-339.58295	0.00
**7**	-339.55387	-339.50465	40.83	-339.56371	-339.51428	43.09
**9 cZc**	-339.63064	-339.58667	-10.64	-339.63892	-339.59369	-6.74
**9 cZt**	-339.63707	-339.59139	-13.61	-339.64494	-339.59936	-10.30
**9 tZc**	-339.62786	-339.58327	-8.51	-339.63928	-339.59444	-7.21
**9 tZt**	-339.63230	-339.58740	-11.10	-339.64388	-339.59891	-10.01
**10**	-339.59021	-339.53967	18.85	-339.59960	-339.54895	21.33
**11**	-339.61189	-339.56157	5.11	-339.62073	-339.57034	7.91
**TS27**	-339.51192	-339.46294	67.00	-339.52675	-339.47758	66.12
**TS79**	-	-	n.a.	-339.56364	-339.51449	42.96
**TS1011**	-339.58963	-339.53983	18.75	-339.59919	-339.54921	21.17

[a] Energy in Hartree. [b] Energy with thermal corrections (298.15 K) in Hartree. [c] Free energy difference in kcal/mol.

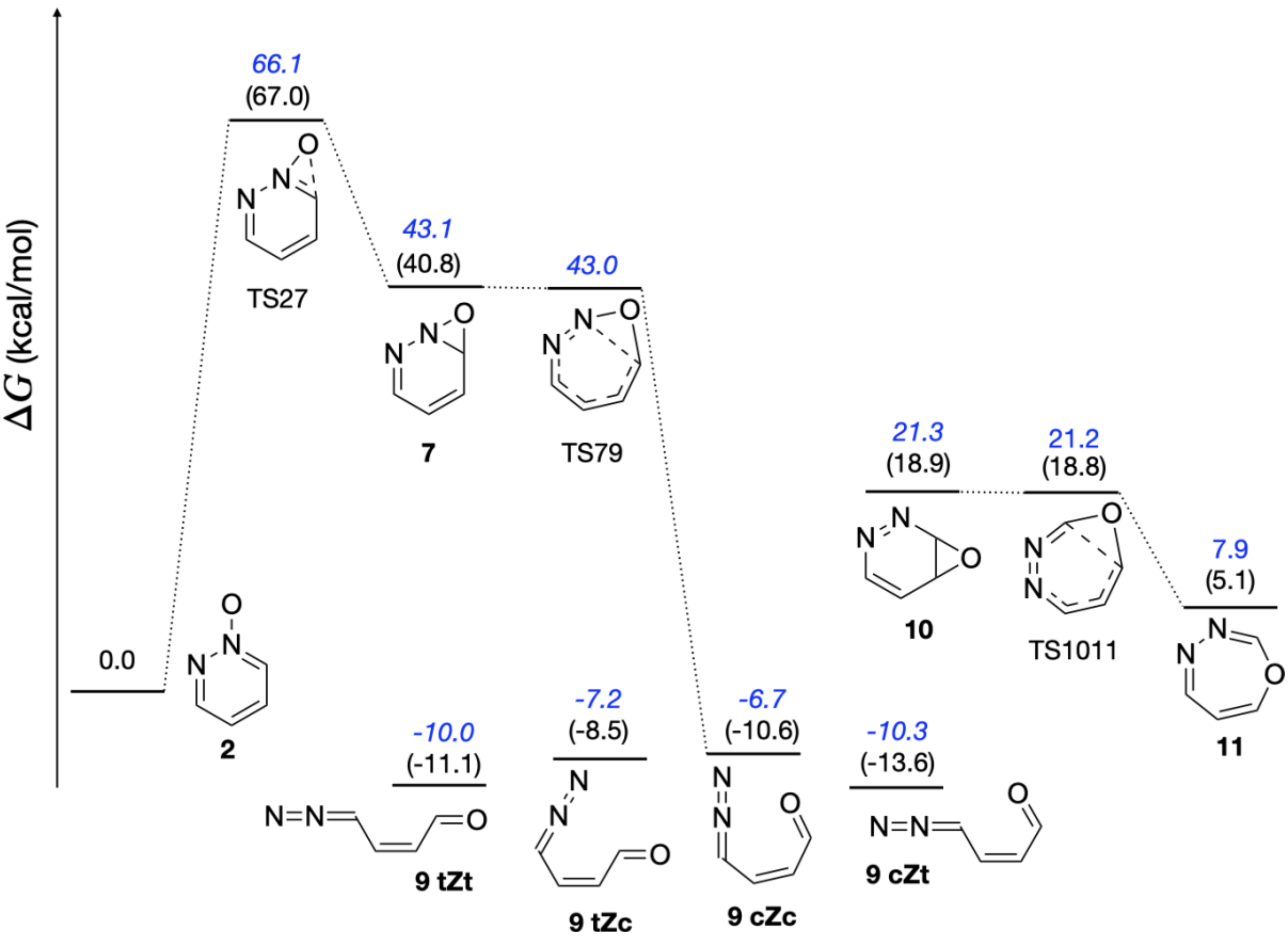

Figure S1. Energy diagram for pyridazine N-oxide **2** and its products computed at the B3LYP/def2-QZVP level. Numbers in blue (and italic) are vibrationally corrected energies using PCM water, numbers in black (in parentheses) without solvent correction.

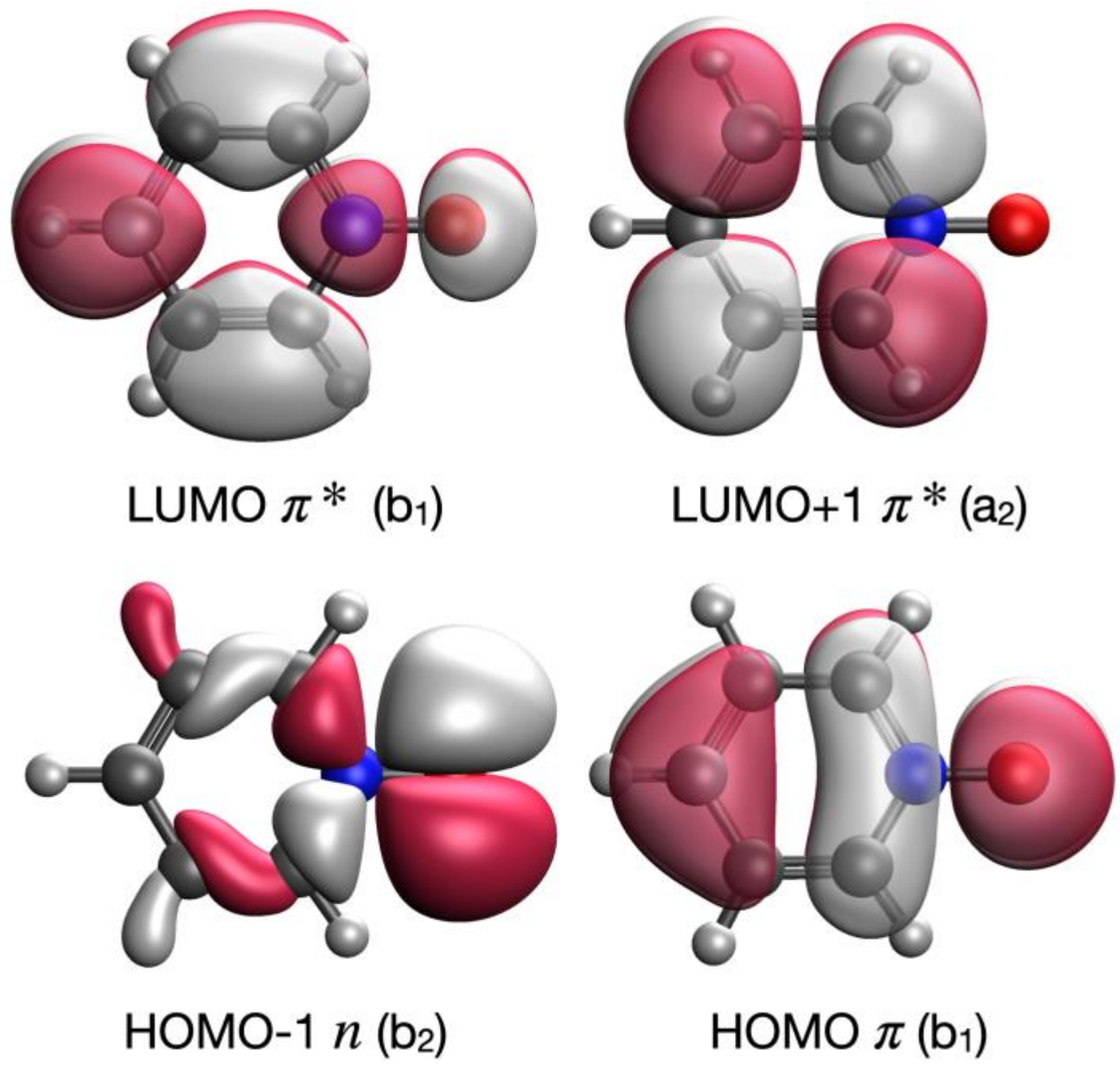

Figure S2. Frontier Molecular Orbitals of pyridine N-oxide **1** (B3LYP/Def2QZVP, isolated molecule)

Excited states of 1.

The excitation energies of the lowest singlet nπ* and ππ* states of **1** were calculated using TDDFT, see Table S3. We compare the isolated molecule, the molecule in dielectric continuum water (Polarizable Continuum Model), both optimized at the B3LYP/def2-QZVP level, and a hydrated pyridine N-oxide. We constructed the latter model as a snapshot from a short molecular dynamics simulation of **1** in water (AMS2024,[1] Universal Force Field), initially keeping a layer of water molecules within 300 pm from the pyridine N-oxide. After a partial optimization (B3LYP/def2-TZVP, polarizable continuum model for water) only a cluster of 5 water molecules that forms a hydrogen-bonded network with the oxygen atom was retained and the energy of the complex was further minimized (See Figure S3). For the TDDFT calculations the def2-QZVP basis set was used.

Table S3. TDDFT excitation energies E (eV) and oscillator strengths f for **1**. Isolated and continuum-solvated structures optimized with B3LYP/def2-QZVP, hydrated structure optimized with B3LYP/def2-TZVP. TDDFT with def2-QZVP basis set.

B3LYP			CAM-B3LYP		
transition	E	f	transition	E	f
		isolated			
$^1A_2$	4.0672	0.0000	$^1A_2$	4.3546	0.0000
$^1B_2$	4.1960	0.0183	$^1B_2$	4.4370	0.0186
$^1A_1$	4.7560	0.2169	$^1A_1$	4.7725	0.2248
		PCM water			
$^1A_2$	4.3748	0.0000	$^1B_2$	4.7012	0.0106
$^1B_2$	4.4136	0.0146	$^1A_2$	4.7582	0.0000
$^1A_1$	4.7128	0.2645	$^1A_1$	4.7849	0.2668
		hydrated			
ππ*	4.8258	0.0035	ππ*	5.0976	0.0328
ππ*	4.9099	0.2870	ππ*	5.1165	0.2629
nπ*	4.9993	0.0137	nπ*	5.5284	0.0010

The results show that the nπ* state is the lowest for the isolated molecule. The continuum model of water changes the energies somewhat, but with the explicit solvent (surrounded by the continuum) the ππ* states are the lowest.

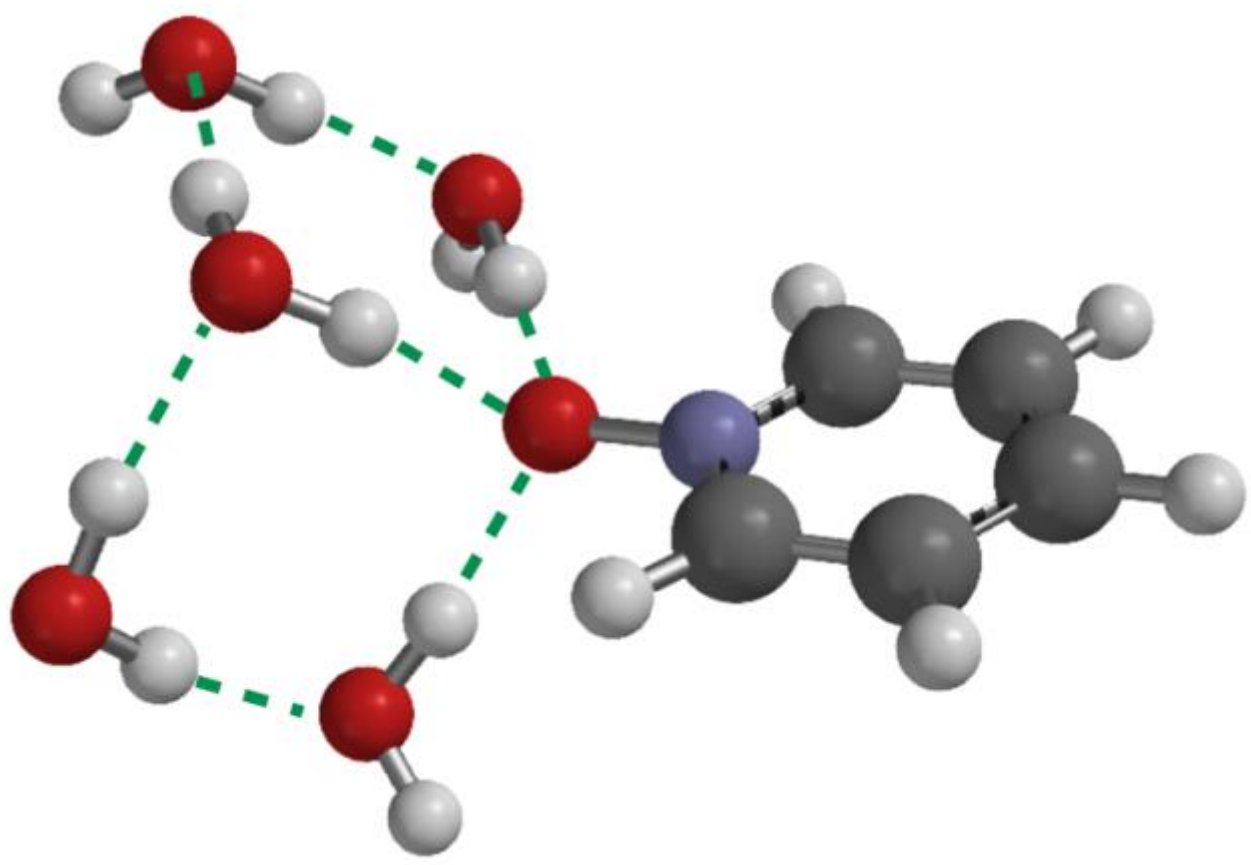

Figure S3. Computed structure of pyridine N-oxide **1** with a hydrogen bonding network. Optimized at the B3LYP/def2-TZVP level, solvated with PCM water. E = -705.991839 E_h.

Geometry optimization of the triplet state (DFT) and the excited singlet state (TDDFT) led to an out-of-plane bending of the O atom in all cases. Due to transfer of charge density from the O atom to the ring, hydrogen bonds in the S_1 state were weaker, and geometry optimization of the cluster of **1** with 5 water molecules became tedious. Therefore, we only retained two water molecules in the final calculation and additionally used PCM without the explicit water molecules. Results are shown in Table S4. It is noteworthy that the isolated S_1 and T_1 states have c_s symmetry and are less bent than the ones using solvation. The TDDFT optimized S1 structure (using PCM water) resembles the MECP structure (CAS-PT2), and the transition structure between **1** and **3** on the ground state energy surface.

Table S4. Optimized structures of excited states of pyridine N-oxide **1**. For comparison, the transition structure linking **1** and oxaziridine **3** is included.

	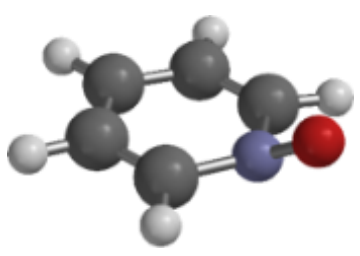	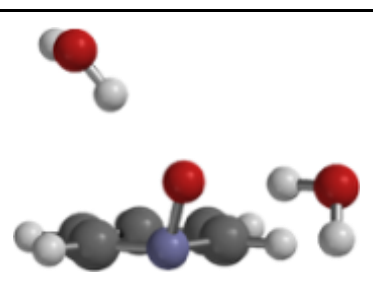	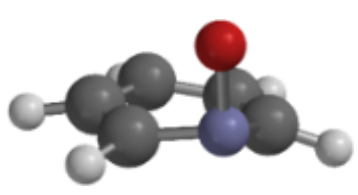	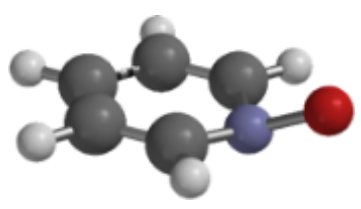	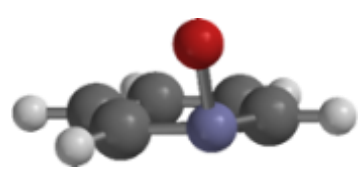	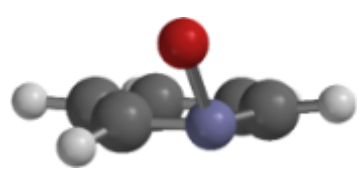
	T_1 (PCM;c_s)	S_1 hydrate	S_1 (PCM)	S_1 isolated (c_s)	MECP	TS **1**→**3** (PCM)
∠C4NO	143.3	117.9	98.0	151.1	105.6	108.3
∠C6NO	115.6	98.1	95.3	113.5	89.7	79.2
E (E_h)	-323.518915	-476.402481	-323.464287	-323.465067		-323.50952

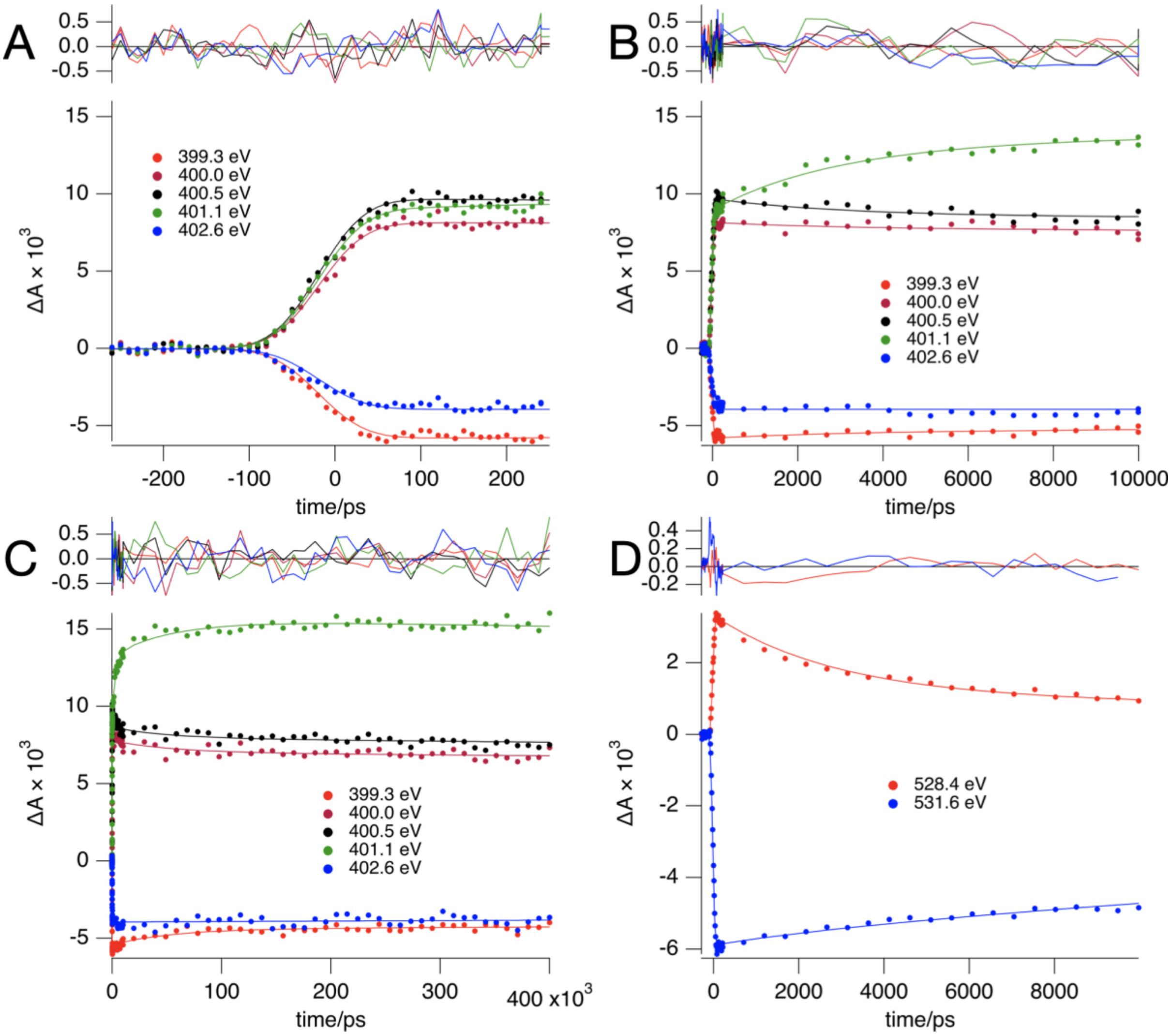

Figure S4. Time-resolved absorption difference of pyridazine N-oxide **2** at the indicated energies, including global fits and residuals, following 258 nm excitation. A-C. at the nitrogen K-edge (data from the same experiments shown at different timescales). D. at the oxygen K-edge. Fit parameters are given in Table 2 in the main text.

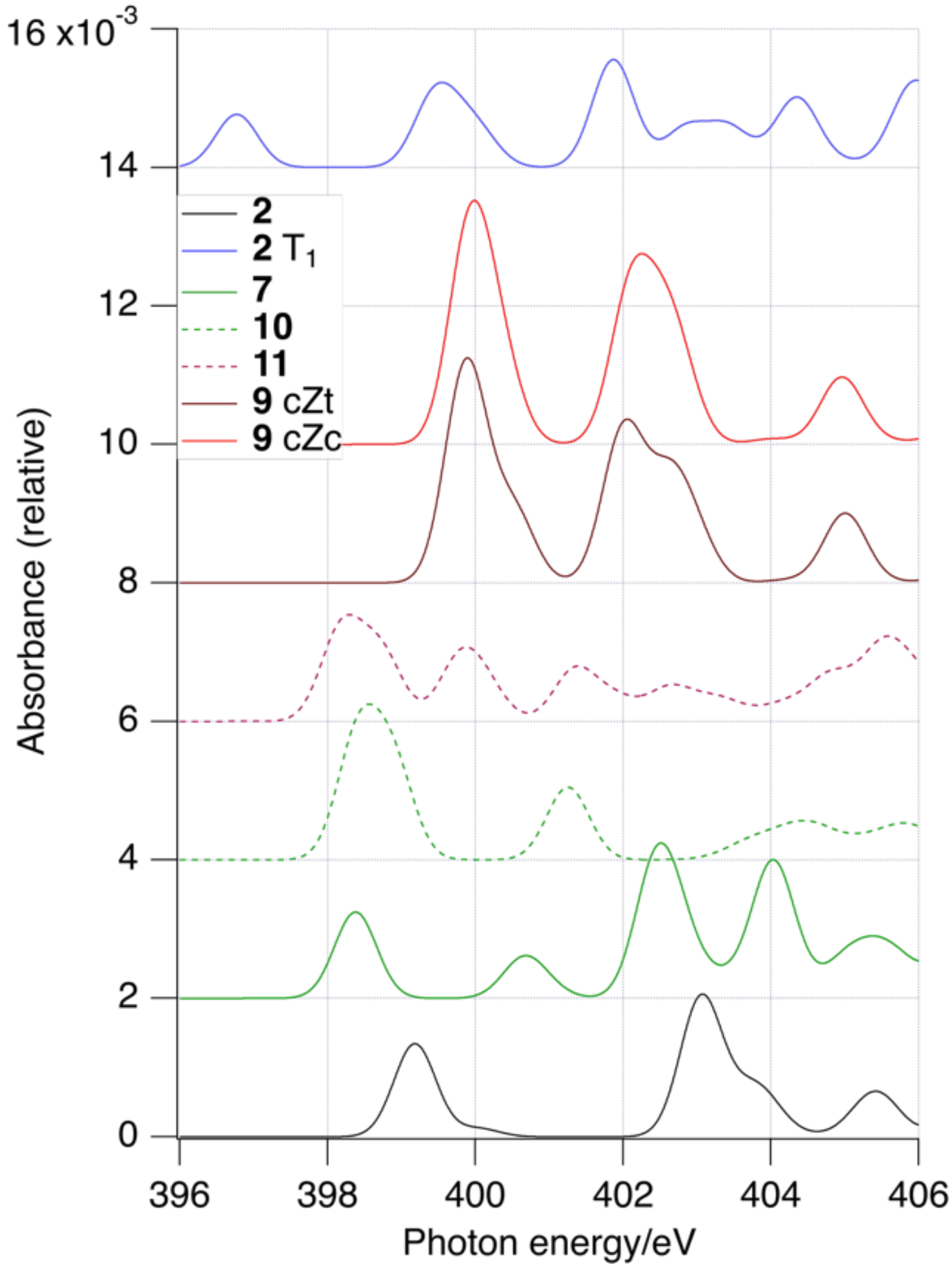

Figure S5. Computed XAS for **2** and its isomers (structures in Figure 1 in the main text). Selected difference spectra are compared with experiment in Figure 11 in the main text.

Table S5. 1s – LUMO excitation energies (eV) calculated with the ΔSCF method (SCAN/def2-QZVPD).[2] Experimental values in parentheses.

	N1	N2	O
**1**	402.18 (402.2)		531.22 (532.2)
**2**	400.26 (399.5)	402.74 (402.8)	531.13 (531.7)
**6**	398.55 (399.0)		
**7**	398.22	403.57	535.54
**9** cZt	401.17	403.57	533.25

References

[1] AMS2024, Scientific Computing and Modeling, Amsterdam, The Netherlands.

[2] S. Jana, J. M. Herbert, Fractional-Electron and Transition-Potential Methods for Core-to-Valence Excitation Energies Using Density Functional Theory, *J. Chem. Theory Comput.* **2023**, *19*, 4100-4113.
